# Successful Treatment of a Severe Case of Fournier's Gangrene Complicating a Perianal Abscess

**DOI:** 10.1155/2011/702429

**Published:** 2011-01-20

**Authors:** Ioannis Papaconstantinou, Anneza I. Yiallourou, Nicolaos Dafnios, Irini Grapsa, George Polymeneas, Dionysios Voros

**Affiliations:** ^1^2nd Department of Surgery, Aretaieion Hospital, University of Athens, 11528 Athens, Greece; ^2^Department of Nephrology, Aretaieion Hospital, University of Athens, 11528 Athens, Greece

## Abstract

A 67-year-old male patient with diabetes mellitus and nephritic syndrome under cortisone treatment was admitted to our hospital with fever and severe perianal pain. Upon physical examination, a perianal abscess was identified. Furthermore, the scrotum was gangrenous with extensive cellulitis of the perineum and left lower abdominal wall. Crepitations between the skin and fascia were palpable. A diagnosis of Fournier's gangrene was made. He was treated with immediate extensive surgical debridement under general anesthesia. The patient received broad-spectrum antibiotics, and repeated extensive debridements were performed until healthy granulation was present in the wound. Due to the fact that his left testicle was severely exposed, it was transpositioned into a subcutaneous pocket in the inner side of the left thigh. He was finally discharged on the 57th postoperative day. Fournier's gangrene is characterized by high mortality rates, ranging from 15% to 50% and is an acute surgical emergency. The mainstay of treatment should be open drainage and early aggressive surgical debridement of all necrotic tissue, followed by broad-spectrum antibiotics therapy.

## 1. Introduction

Fournier's gangrene (FG) is a rare, synergistic, fulminant form of necrotizing fasciitis involving the genital, perineal, and perianal regions [1]. After first description, understanding of this disease has considerably changed and its epidemiology, clinical features, and pathogenesis have been well defined. 

Nowadays, FG is defined as a potentially fatal condition, affecting any age and gender, which results in thrombosis of small vessels, obliterative endarteritis, and eventually skin and tissue necrosis [2]. Predisposing factors believed to contribute to the development of the disease are diabetes mellitus, alcoholism, malignancies, immunosuppression, lever, and renal disease [3–5]. In the majority of cases, aerobic and anaerobic bacteria are synergistically involved as a result of anorectal and urogenital trauma and/or infection. 

FG is characterized by high mortality rates, ranging from 15% to 50% and is an acute surgical and urological emergency [1]. The keystones of management are hemodynamic stabilization, effective antibiotic treatment, and urgent aggressive surgical debridement.

We present a case of Fournier's gangrene following a perianal abscess which was successfully treated in our department despite the severity of the patient's condition.

## 2. Case Presentation

**Figure 1:**
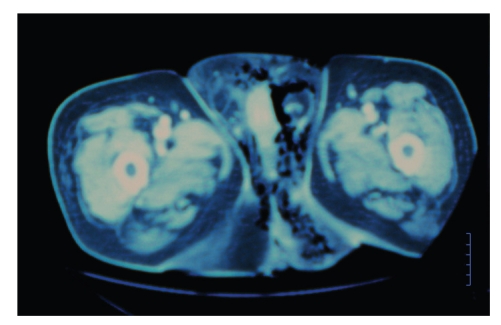
Computed tomography of the pelvis dipecting extensive emphysema around the testicles and perineal subcutaneous tissue.

**Figure 2:**
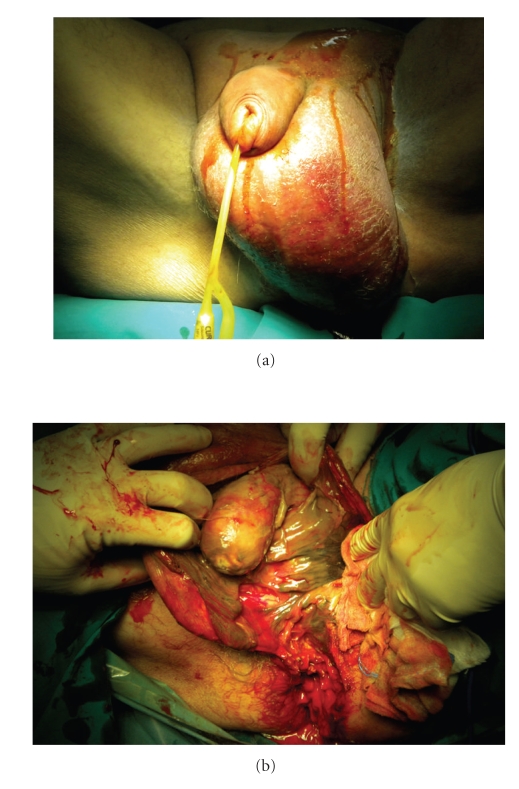
(a) Preoperative image showing considerable edema and scrotal necrosis. (b) Intraoperative image of the immediate surgical debridement demonstrating the extensive necrosis of the scrotum and perianal region.

**Figure 3:**
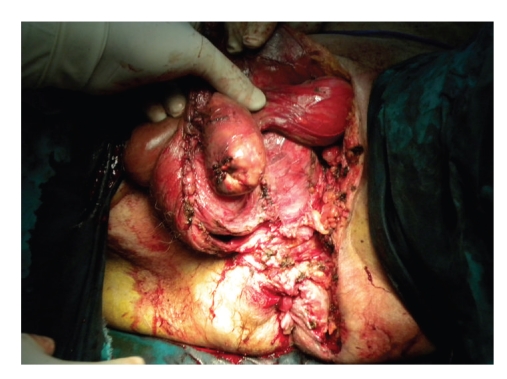
Wide-open drainage of the necrotic skin in the scrotum and perineal area.

A 67-year-old male patient was admitted to our hospital with severe perianal pain and high fever (39°C). The patient was obese and had a history of diabetes mellitus under oral hypoglycemics and nephritic syndrome due to glomenulonephritis under cortisone treatment. Upon physical examination, the patient was hypotensive (90/60 mm Hg) and tachycardic (120/min). A perianal abscess was identified. Furthermore, the scrotum was gangrenous with extensive cellulitis of the perineum and left lower abdominal wall. Crepitations between the skin and fascia were palpable. White blood cell count was 10000/*μ*L, C-reactive protein (CRP) was 25 mg/dL, and blood glucose was 300 mg/dL. Computed tomography of the lower abdomen and pelvis revealed extensive emphysema around the testicles, perineal subcutaneous tissue, and around the left internal iliac vessels (Figure 1). A diagnosis of Fournier's gangrene complicating a perianal abscess was made. The patient underwent aggressive fluid administration and hemodynamic support. He was treated with immediate extensive surgical debridement under general anesthesia (Figures 2(a) and 2(b)). Tissue cultures were obtained for the isolation of the responsible microorganisms. The necrotic skin in the scrotum and the perianal region was evacuated into a wide-open drainage area, without any damage to the testicles spermatic cords, or external sphincter (Figure 3). In addition, a diverting colostomy was performed. 

Preoperative antibiotic treatment with broad-spectrum antibiotics combinations was initiated and later adjusted to the culture sensitivity of the microbial isolates. According to tissue samples taken during debridement, the microbiological etiology of Fournier's gangrene was polymicrobial. Staphylococcus haemolyticus, Citrobacter braakii, and Morganella morganii were detected. Therefore, the patient received metronidazole, penicillin G, ciprofloxacin, and teicoplanin intravenously. Regarding nutrition, he initially received total parenteral nutrition. We continued with enteral nutrition on the 5th postoperative day, and finally he received oral feeding a few days later.

**Figure 4:**
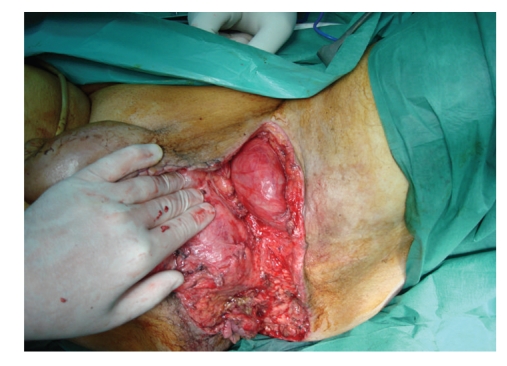
The left testicle is transpositioned into a subcutaneous pocket in the inner side of the left thigh.

He underwent three subsequent surgical debridements. Due to the fact that his left testicle was severely exposed, it was transpositioned into a subcutaneous pocket in the inner side of the left thigh on the 7th postoperative day (Figure 4). Local wound care was performed with moist gauze dressings (i.e., normal saline) changed twice daily until healthy granulation tissue was observed. Subsequently, dry dressings were used. 

**Figure 5:**
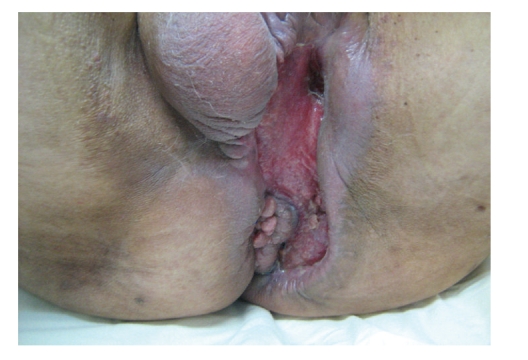
Image of the surgical wound four weeks after the initial operation showing that the gas gangrene has completely resolved, the wide wound is reduced, and good granulation is present.

The infection gradually subsided, the gas gangrene resolved completely, and good granulation was present four weeks after surgery (Figure 5). The viability of the testicle was confirmed with a Doppler ultrasound on the 50th postoperative day. With regards to the diabetes mellitus, the blood glucose levels remained within normal levels with oral hypoglycemic as the only treatment. The patient was finally discharged on the 57th postoperative day.

## 3. Discussion

The syndrome of FG is an uncommon but quite serious problem. This entity affects both men and women and at a wide age range, from neonates to the very elderly. Despite this, the mean age of patients appears to range from 40 to 50 years [1]. Our patient was 67 years old, which is in accordance to some recent reports of an increase in the peak age incidence.

Earlier, FG was considered to be an idiopathic entity, but nowadays the most common initial ports of entry are thought to be local trauma or extension of a urinary tract or perianal infection [6]. With regards to the genitourinary tract, urethral strictures and transurethral instrumentation are the most frequent etiologies; other causes include surgery of the penis and scrotum, transrectal prostate biopsy, urethral calculi, bladder cancer infiltrating the urethra, and phlebitis of dorsal penis vein [7–9]. Anorectal sources of infection include ischiorectal, perianal, and intersphincteric abscesses, especially those inadequately treated [8]. Diverticular perforation, carcinoma of the sigmoid colon and rectum [10], perforated acute appendicitis [11], internal hemorrhoids ligated with rubber bands [12], and anal dilatation [13], have also been reported in the etiology of FG. In our patient, a perianal abscess was found to be cause of FG.

The infection arises from bacteria inoculation in the perineal area. This procedure can be facilitated by an impairment in the immune system; diabetes mellitus, alcoholism, malignancies, leukemia, treatment with steroids, AIDS, renal failure, and hemodialysis predispose to the development of FG [3–5]. Diabetes mellitus, in particular, represents an apparent associated factor due to the defective phagocytosis, the increased incidence of urinary tract infections as a result of functional urinary tract obstructions from diabetic neuropathy and disease of the small vessels [5]. In our case, the patient suffered from diabetes mellitus and obesity and was treated with cortisone due to glomerulonephritis.

The most common clinical features are perianal pain and swelling if the anorectal area is the portal of entry, whereas urinary retention, testicular, or scrotal pain are present if the infection launches from the genitourinary tract [8]. Other systemic manifestations such as fever, tachycardia, electrolyte imbalances, and hyperglycemia may also be present. Our patient was admitted to our hospital with severe perianal pain, scrotal edema, crepitus, fever, tachycardia, and low blood pressure.

Once a diagnosis of FG has been established, the central principles of management are aggressive hemodynamic stabilization and parenteral broad-spectrum antibiotics which will be either changed or continued according to the culture findings. In order to ensure a successful outcome, the critical step is urgent and extensive surgical debridement [1, 2, 7]. All frankly necrotic tissue and those with doubtful viability should be carefully debrided and excised. 

Colostomy should be done in selected cases where the FG involves the anorectal area and sphincter and for patients in high risk of fecal contamination. We decided to perform a diverting colostomy in our patient, since the gangrene extended to perianal area and fecal contamination would therefore be expected. 

Testes and spermatic cords are generally not affected by this disease, because they are supplied by the testicular artery. In some studies, patients underwent orchiectomy when severe infection in the peritesticular tissues was observed intraoperatively [2]. In our case, the left testicle was totally exposed, so it was placed subcutaneously in the inner side of the left thigh. Although, concerns over temperature regulation, future function of the testicles, testicular pain, and atrophy have been expressed [14], none of these problems were encountered in our case. Moreover, the viability of the testicle was confirmed by Doppler ultrasound several days postoperatively.

Although the number of patients with FG has decreased due to medical progress, the mortality is still high. In patients presenting with sepsis, diabetes mellitus, and late admissions to the hospital mortality rates were found to be highest [4]. Hospitalization for this disease is extremely long with a reported average of six weeks [15]. Our patient survived and was discharged 57 days after admission to the hospital, despite the severity of his condition and the negative prognostic factors. 

## 4. Conclusions

Fournier's gangrene represents a severe condition with a high morbidity and mortality. Therefore, an aggressive multidisciplinary management is mandatory. Fluid resuscitation, antibiotic therapy, nutritional support and, most importantly, repeated surgical debridement remain the cornerstone of the therapeutic approach.
